# Determinants and risk factors for renal damage: where do patients hospitalized for severe anorexia nervosa stand? A multi-center study

**DOI:** 10.1186/s40337-024-01024-w

**Published:** 2024-06-05

**Authors:** Chantal Stheneur, Corinne Blanchet, Lama Mattar, Marika Dicembre, Kayigan Wilson, EVHAN Group, Jeanne Duclos, Hélène Roux, Marie-Raphaële Thiébaud, Sarah Vibert, Tamara Hubert, Annaig Courty, Damien Ringuenet, Jean-Pierre Benoit, Marie-Rose Moro, Laura Bignami, Clémentine Nordon, Frédéric Rouillon, Solange Cook, Catherine Doyen, Marie-Christine Mouren, Priscille Gerardin, Sylvie Lebecq, Marc-Antoine Podlipski, Claire Gayet, Malaika Lasfar, Marc Delorme, Xavier Pommereau, Stéphanie Bioulac, Manuel Bouvard, Jennifer Carrere, Karine Doncieux, Sophie Faucher, Catherine Fayollet, Amélie Prexl, Stéphane Billard, François Lang, Virginie Mourier-Soleillant, Régine Greiner, Aurélia Gay, Guy Carrot, Sylvain Lambert, Morgane Rousselet, Ludovic Placé, Jean-Luc Venisse, Marie Bronnec, Bruno Falissard, Christophe Genolini, Christine Hassler, Jean-Marc Tréluyer, Olivier Chacornac, Maryline Delattre, Nellie Moulopo, Christelle Turuban, Christelle Auger, Sylvie Berthoz, Mouna Hanachi, Nathalie Godart

**Affiliations:** 1 University Center for Adolescent and Young Adult Health, Fondation Santé des Etudiants de France, 75014 Paris, France; 2 CESP, INSERM, UMR 1018, University Paris-Sud, 94807 Villejuif Cedex, France; 3 UVSQ, UFR Simone Veil, University Paris-Saclay, Montigny le Bretonneux, France; 4 Maison de Solenn-Maison des Adolescents, Cochin Hospital, Assistance Publique-Hôpitaux de Paris, 75014 Paris, France; 5 Nutrition Program, Department of Natural Sciences, School of Arts and Sciences, Lebanese American University, Beirut, Lebanon; 6 Univ. Bordeaux, INCIA CNRS UMR5287, 33000 Bordeaux, France; 7 Department of Psychiatry, Institut Mutualiste Montsouris, 75014 Paris, France; 8 Clinical Nutrition Unit, Paul Brousse University Hospital, Assistance Publique-Hôpitaux de Paris, Villejuif, France; 9 Paris-Saclay University, INRAE, AgroParisTech, Micalis Institute, Jouy-en-Josas, France

**Keywords:** Anorexia nervosa, Renal damage, Renal function, Kidney functionality tests, Body mass index

## Abstract

**Background:**

Although renal damage is increasingly reported among the most undernourished patients with Anorexia Nervosa (AN), it remains underestimated in current practice, and often associated with acute dehydration. The purpose of our study was to evaluate the frequency, the extent, and the risk factors of renal involvement among adolescents and adults hospitalized in specialized units for AN.

**Methods:**

In this multi-center study, 197 consecutive participants were included, aged 13–65, from 11 inpatient eating disorder psychiatric units. Information on the course of AN, clinical characteristics, biological data, and medication were collected.

**Results:**

At admission, mean BMI was 13.1 (± 1.6) kg/m^2^ for a mean age of 20.74 (± 6.5) years and the z-score was − 3.6 (± 1.33). Six participants (3.0%) had hyponatremia, four (2.0%) had hypokalemia, and nine (4.5%) had hypochloremia. The Blood Urea Nitrogen/Creatinine ratio was over 20 for 21 (10.6%) participants. The mean plasma creatinine was 65.22 (± 12.8) µmol/L, and the mean eGFR was 74.74 (± 18.9) ml/min. Thirty- five participants (17.8%) had an eGFR > 90 ml/min, 123 (62.4%) from 60 to 90 ml/min, 35 (17.8%) from 45 to 60 ml/min, and 4 (2%) under 45 ml/min. In multivariate analysis, only BMI on admission was a determinant of renal impairment. The lower the BMI the more severe was the renal impairment.

**Conclusion:**

When eGFR is calculated, it highlights renal dysfunction found in severe AN requiring hospitalisation in specialized units. The severity of undernutrition is an independent associated factor. Kidney functionality tests using eGFR, in addition to creatinine alone, should be part of routine care for patients with AN to detect underlying renal dysfunction.

## Abstract (plain language summary)

AN is a psychiatric illness with organic repercussions that are not always visible nor frequently investigated. Renal damage, if detected, is often attributed to dehydration, and is thought to be rapidly reversible. Assessment of its severity and evolution is therefore not systematic, even in eating disorder units specialised in the care of patients with AN. Our study explored the assessment of renal impairment among adolescents and adults hospitalized in psychiatric units using eGFR calculation. Our results showed that fewer than 18% of the patients hospitalized had normal renal function and that among the various criteria, only BMI on admission was related to the extent of this impairment. Assessment of renal function by eGFR calculation and not only by creatinine measures should be performed routinely in all situations requiring hospitalization in anorexia nervosa, regardless of the reason for hospitalization.

## Background

Anorexia Nervosa (AN) is an eating disorder characterized by refusal to maintain normal body weight and intense fear of gaining weight or becoming obese, coupled with a severely disturbed body image [1]. AN patients generally restrict their calories, often accompanied by compulsive exercising, excessive vomiting and abusive use of diuretics and laxatives to control their weight [2]. Two subtypes are defined as followed: (1) Restricting type: Over the previous 3 months, the individual did not engage in recurrent episodes of binge eating or purging behaviours (i.e. self-induced vomiting or the misuse of laxatives, diuretics, or enemas). This subtype includes presentations in which weight loss is accomplished primarily through dieting, fasting, and/or excessive exercise; (2) Binge-eating/purging type: over the previous 3 months, the individual engaged in recurrent episodes of binge-eating or purging behaviours (i.e. self-induced vomiting or the misuse of laxatives, diuretics, or enemas) [1]. Although primarily a mental health disorder, AN has numerous serious somatic consequences, and it is associated with high rates of morbidity and mortality [3]. The continuous reduction of energy intake by patients with AN induces metabolic changes leading to different multi-organ complications, such as cardiovascular and liver disturbances, neuroendocrine dysfunction, and in some cases specific nutritional deficiencies [4]. These consequences are not only immediate but can also have sequalae that can persist for years. Steinhausen reported the hazard ratio for any somatic disease for patients compared to controls increased around the time of admission to hospital, with increments ranging from 14% for respiratory system disease to 300% for circulatory system diseases, and then declined to under 1.50% in the longer term [5]. While osteoporosis remains the most frequent, documented, long-term complication, other complications such as endocrine, dental or urological problems can also persist [6–8]. Furthermore, compensatory behaviours such as excessive vomiting and abuse of diuretics can lead to severe electrolyte disturbances, alterations in water metabolism, and severe hypokalaemia [9]. Acute and chronic kidney disease (CKD) have been previously described in AN [10–13]. The main factors predicting low levels of renal filtration at admission are the degree of bradycardia and weight loss or low BMI [14]. Over 70% of subjects with AN (independently from the subtypes AN-R or BP) have renal complications at some point in their lives [15], with a 5.2% prevalence of severe kidney disease, including terminal renal failure after 21 years of AN [13]. Recently, a cohort study from Taiwan reported that when compared with a control group, 2091 out of over 43,951 individuals had AN and a significantly higher risk of requiring acute dialysis (adjusted hazard ratio 2.10 [95% confidence interval 1.19–3.68]), hypokalaemia, hypovolemia, nephritis, acute renal failure, and chronic renal failure, but no significantly higher risk of end-stage renal disease [16]. The mortality study by Fichter et al. [17] found that kidney failure was the cause of death for 2 out of 31 AN patients who died from natural causes. Although it is increasingly reported, kidney damage remains underestimated in current practice, with teams unfamiliar with kidney damage often contenting themselves with falsely normal creatinine levels, without calculating the eGFR. Diagnosis of renal disease should involve more than just measuring blood creatinine, because patients who are undernourished as a result of AN often have very low muscle mass and their dietary intake of protein is often very inadequate [18].

Thus, the aim of this study in a large cohort of adolescents and adults hospitalized for AN in 11 different centres in somatic or psychiatric care was to evaluate the frequency, extent, and severity of renal involvement using the estimated glomerular filtration rate (eGFR) at admission and factors related to the decreases in eGFR.

## Material and methods

This study is a part of a large cross-sectional multi-centre study named EVHAN (Evaluation of Hospitalization for AN, Eudract number: 2007-A01110-53, registered in Clinical trials).

Subjects were recruited from the inpatient treatment facilities of 11 centres across France (see EVHAN group for details). Two hundred and forty-two consecutive participants with AN were recruited in the EVHAN study from April 2009 to May 2011.

Inclusion criteria for the current study were as follows: being hospitalized for AN, admission BMI < 15 kg/m^2^ and/or sudden and rapid weight loss, agreement to participate in the study, and being affiliated to the French Social Security health coverage system. Exclusion criteria were refusal to participate, insufficient understanding of the French language, the existence of a potentially confounding pathology (e.g. diabetes, Crohn’s disease or other metabolic disorders) and being under the age of 13. For this study, because of the possible difference in muscle mass and serum creatinine levels between males and females, we decided to limit our investigation to females.

The diagnosis of AN was based on DSM-IV-TR criteria using the Eating Disorder Examination (EDE-Q v. 5.2) and the CIDI 3.0, with the following criteria for BMI: < 10th percentile up to 17 years of age, and BMI < 17.5 kg/m^2^ for 17 years of age and older [19].

Participants were assessed by questionnaire and enrolled in the study in the first two weeks of hospitalization, but the biological tests were usually carried out within the first day of admission, as is the case in routine practice.

The 11 centres were all units specialized in the clinical management of patients with AN.

The data collected was divided into four categories:

- Information relating to the course of AN, including minimum and maximum weight and body mass index (BMI = weight (kg)/height (m)^2^), duration of disease, and pubertal status (pre- or post- menarche). Because normative BMI values vary with age among adolescents, and in order to compare adolescents and adults, all BMI values were converted into z-scores [20]. Delta BMI was calculated as the maximum lifetime BMI minus the minimum lifetime BMI. It is expressed in kg/m^2^ but also as a z-score (delta z-score) to take account of age and development.
- Clinical characteristics at admission: age (years), weight (Kg), height (m), current BMI (kg/m^2^ and z-score), eating disorder symptoms (EDE-Q questionnaire), duration of illness, number of previous hospitalizations, and psychotropic medication taken at the time of hospitalization, whether these medicines were prescribed by doctors or purchased over the counter. Use of medication: Drugs prescribed for therapeutic purposes were listed not for their nephrotoxicity but as markers of the probable severity of the disease. Drugs used with or without prescription, such as laxatives, were listed on the basis of patient report.
- The Eating Disorder Examination Questionnaire (EDE-Q) is a 28-item self-reported questionnaire adapted from the semi-structured interview Eating Disorder Examination (EDE) and designed to assess the range and severity of features associates with a diagnosis of eating disorder using 4 subscales (Restraint, Eating Concerns, Shape Concerns and Weight Concerns) and a global score [21].

Our sample included subjects hospitalized for AN diagnosed by clinicians using the DSMI-V R-criteria. Evaluations using our standardized method found both full and sub-threshold AN (sAN) (for details about the sAN sub-sample see Rizk et al. [22]. Hereafter these profiles are referred to as AN. We retrieved and described them according to the sub-type with the distinction between the restrictive sub-type (AN-R) and the binge/purging sub type (AN-BP)

Usual biological data was obtained on the day of admission: concentration of blood electrolytes (Na, K, Cl,), blood urea nitrogen (BUN) levels, and plasma creatinine levels. The Estimated Glomerular filtration rate (eGFR) was calculated using the Cockroft–Gault Formula: (eGFR = (140 − age) × mass (in kilograms) × [0.85 if female] / 72 × serum creatinine (in mg/dL) (Drinka PJ., 1989). In this study, we chose the Cockroft and Gault equation, as this equation includes weight, and has been shown to correlate optimally with measured glomerular filtration rates [23, 24]. In the literature, Chronic Kidney Disease (CKD) is characterized as follows: stage 1 normal or high GFR (GFR > 90 mL/min), stage 2 mild CKD (GFR = 60–89 mL/min), stage 3 mild to moderate CKD (GFR = 45–59 mL/min), and stage 3B moderate to severe CKD (GFR = 30–44 mL/min) in line with the KDIGO working group guidelines (Kidney Disease Improving Global Outcomes CKD Working Group, 2013). Although our population did not present chronic renal failure, we decided to retain the same glomerular filtration thresholds

## Statistical analyses

Demographic and clinical characteristics of participants were described in the overall sample using numbers and proportions for categorical variables and means and standard deviations (SD) for continuous variables. We first used bivariate logistic regression to assess the independent association between renal dysfunction and other clinical/and demographic factors. For bivariate analyses, we included the following variables: age, BMI (z-score), Delta BMI, duration of disease, pubertal status, diagnosis and sub-type, number of previous hospitalizations, EDEQ score, use of medicines, and biological data. In the multivariate model we included all the variables associated with renal dysfunction with a *p*-value < 0.1 in bivariate analysis.

To determine the factors influencing the decrease in eGFR, we divided the study sample into two groups: those with little or no renal damage (stages 1 or 2) and those with moderate to severe damage (stages 3 A or 3 B).

All analyses were performed using R Statistical Software (v4.1.2; R Core Team 2021). A two-sided *p*-value < 0.05 was considered statistically significant.

## Procedure and ethical considerations

This study was part of the larger, longitudinal, multi-centre study, EVHAN (Evaluation of Hospitalization for AN, Eudract number: 2007-A01110-53, registered on Clinical trials). The study protocol was approved by the Ile-de-France III Ethics Committee and the CNIL (Commission Nationale de l’Informatique et des Libertés). Written informed consent was obtained from each patient before inclusion (adults; minors and their parents). All methods were carried out in accordance with relevant guidelines and regulations.

## Results

Two hundred and twenty-two participants (with full syndrome or sub-threshold AN) were included in this study. Out of these 222, 15 participants were excluded because of missing data, 10 were men, and therefore the remaining 197 women participants were included in the present study.

### Clinical and biological characteristics at admission

The clinical and biological characteristics at admission are presented in Table 1. The average age at admission was 20.74 years (± 6.5) years; min: 13.16; max: 52.6 years). The average duration of the disease was around 4 years, with an average of almost 3 previous hospitalisations. Nearly 30% of the patients did not meet all the criteria for AN and were therefore classified as sAN. About half the patients reported repeated vomiting. The maximum lifetime BMI was in the normal range (20.3 (± 3.3) kg/m^2^) and the mean BMI at admission was 13.1 (± 1.6) kg/m^2^. The mean difference between the maximum and minimum lifetime BMI was 7.02 (± 3.2) kg/m^2^.

**Table 1:** Clinical and paraclinical characteristics upon admission

Variable	Mean (SD)
Age (years)	20.74 (6.5)
Lowest lifetime BMI (kg/m^2^)	13.1 (1.6)
BMI at admission z score	− 3.6 (1.33)
Delta BMI max – BMI adm z score	2.8 (2.1)
Duration of disease (years)	4.08 (4.25)
Number of previous hospitalizations	2.7 (4.2)
EDE-Q score	3.25 (1.3)
NA MMOL/L	139.1 (3.3)
K, MMOL/L	4.03 (0.5)
CL, MMOL/L	102.1 (4.2)
BUN, MMOL/L	4.7 (1.7)
Variable	N (%)
Diagnostic	
AN	142 (72.8)
sAN	53 (27,2)
Subtype	
AN-R	100 (50.7)
AN-BP	97 (49.3)
Patients with menarche	174 (88.8)
	Number (N (%)) of patient prescribed the below treatments:
Antidepressants	66 (33.5)
Thymoregulators	5 (2.5)
Neuroleptics	27 (13.8)
Hypnotics	33 (16.8)
Anxiolytics	81 (41.1)
Benzodiazepine	52 (26.5)
Laxatives or Diuretics use	24 (12.2)

*AN* Anorexia nervosa, *sAN* sub-thresold Anorexia Nervosa, *AN-BP* Anorexia nervosa binge purging subtype, *AN-R* Anorexia nervosa restrictive subtype

The mean admission BMI for AN-BP was significantly higher than for AN-R (14.7 and 13.8 kg/m^2^ respectively) (*p* < 0.001).

Concerning the biological data (Table 1), 6 (3.0%) participants had hyponatraemia, 4 (2.0%) hypokaliemia, and 9 (4.6%) hypochloraemia. The BUN/Creatinine ratio was over 20 for 21 (10.6%) participants.

Among the psychotropic drugs most frequently used, anxiolytics were found for over 40% of the patients, followed by antidepressants for over 30%.

### Assessment of renal function

**Fig. 1:**
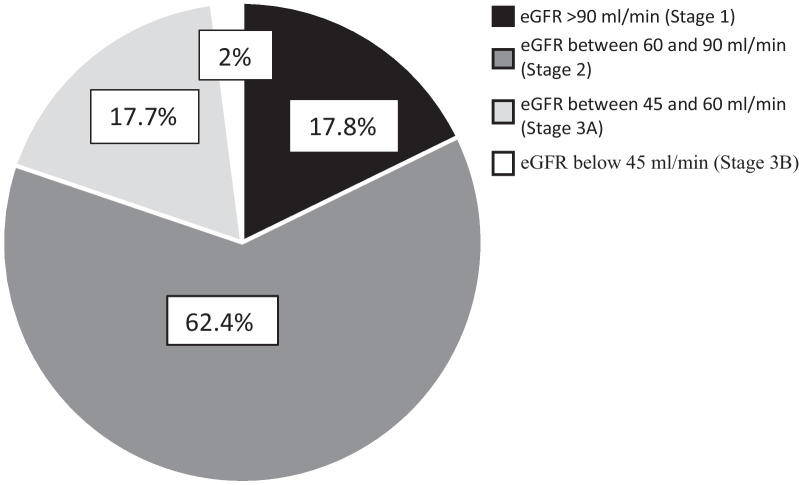
Percentages for different stages of kidney disease assessed by eGFR among participants hospitalized for anorexia nervosa

At admission, the mean plasma creatinine was normal or low 65.2 (± 12.8) µmol/l (min 6.5; max 106). The mean eGFR was 74.7 (± 18.9) ml/min (min: 38.3; max: 168.5) i.e. stage 2 mild CKD. A total of 35 participants (17.8%) had an eGFR > 90 ml/min, 123 (62.4%) from 60 to 90 ml/min, 35 (17.8%) from 45 to 60 ml/min and 4 (2%) under 45 ml/min (Fig. 1).

### Factors associated with decreased eGFR

In bivariate analysis, the factors associated with a decrease in eGFR were the extent of undernutrition, lifetime or at admission, the number of previous hospitalizations, the subtype AN-R and the prescription of antipsychotics (Table 2).

**Table 2:** Mean values and frequencies of clinical factors according to CKD stage (Stage 1 or 2 vs stage 3 or 4)

Variable	eGFR < 60 n = 39	eGFR > = 60 n = 158	*p*
	Mean (SD)		
Age, mean	21. 6 (6.6)	20.5 (6.5)	0.31
BMI z score	− 4.5 (1.2)	− 3.4 (1.3)	< 0.0001*
Delta BMI max –BMI adm	2.9 (2.1)	2.8 (2.2)	0.72
Duration of AN	6.9 (5.4)	5.4 (3.9)	0.09
Number of previous hospitalizations	4.6 (7.4)	2.2 (2.9)	0.01*
EDEQ score	3.3 (1.24)	3.2 (1.32)	0.66

	N (%)	*p*	*P*
*Diagnosis*			
AN	28 (71.8)	114 (73.1)	0.87
sAN	11 (28.2)	42 (26.9)	
*Subtypes*			
AN-BP	15 (38.5)	82 (51.9)	0.135
AN-R	24 (61.5)	76 (48.1)	
Antidepressants	17 (43.6)	49 (31)	0.14
Thymoregulators	2 (5.1)	3 (1.9)	0.37
Neuroleptics	9 (23.1)	18 (11.5)	0.06
Hypnotics	10 (24.6)	23 (14.6)	0.1
Anxiolytics	19 (48.7)	62 (39.3)	0.2
Benzodiazepine	14 (35.9)	38 (24.2)	0.1
Laxatives or Diuretics use	34 (87.2)	139 (88)	0.9
Menarch yes	36 (92.3)	138 (87.9)	0.4

*Statistical significance is set at *p* < 0.05. sub-thresold *AN* Anorexia nervosa, *sAN* sub-thresold anorexia nervosa, *AN-BP* Anorexia nervosa binge purging subtype, *AN-R* Anorexia nervosa restrictive subtype

For participants with an eGFR below 45 ml/min (stage 3B), the mean z-score BMI tended to be lower, although this difference was not significant, than for the overall sample − 6.6 (± 1.5) kg/m^2^ versus − 3.6 (± 1.33), with a decrease of 7.1 ± 0.24 kg/m^2^ between maximum BMI and admission BMI.

In multivariate analysis, only the admission BMI expressed as a z score was shown to be a determinant for renal impairment taking account of duration of AN, number of previous hospitalisations, and psychotropic medication (including neuroleptics, hypnotics, benzodiapines).

The lower the admission z-score BMI, the more severe was the renal impairment (Table 3).

**Table 3:** Factors associated with decreased DFG in multivariate analysis

variable	Odds ratio [95% CI]	*p*
BMI z-score	0.6 [0.42–0.83]	0.003*
Duration of AN	1.01 [0.92–1.11]	0.7
Number of previous hospitalizations	1.05 [0.96–1.14]	0.28
Neuroleptic	1.87 [0.64–5.20]	0.23
Hypnotic	1.94 [0.65–5.58]	0.22
Benzodiazepine	1.14 [0.42–2.14]	0.78

*AN* Anorexia nervosa

*Statistical significance is set at* p* < 0.05

## Discussion

This study shows that at the time of admission most of the subjects hospitalized for severe AN (more than 80% of the participants) presented a serious, marked decrease in the glomerular filtration rate below the threshold of 90 ml/min, with a mean eGFR of 74.74 (± 18.9) ml/min. An eGFR under 60 ml/min (Stage 3A and 3B of CKD) was linked to the extent of undernutrition at admission, independently from the duration of AN, the number of past hospitalizations, or the current psychotropic treatment (including neuroleptics, hypnotics, and benzodiazepines).

Comparisons with other studies on low eGFR values are sometimes difficult because of the different methods used to calculate eGFR. It has previously been shown that glomerular filtration should not be calculated with equations such as MDR, which has little or no correlation with the measured glomerular filtration rate [23, 24].

In adolescent populations (age < 18 years), the six studies evaluating glomerular filtration rate among patients with AN used different measurement methods [10–12, 14, 18, 24, 25]. Three are comparable because the eGFR was calculated with the same equation: the Cockroft and Gault equation. The mean eGFR values were 63.9, 74.0 and 99 ml/min [11, 12, 24]. The first study, which found the lowest eGFR, was conducted on a sample of subjects who were older than in the other studies (16y.o ± 6 months vs. 14y.o ± 9 or 10 months), with a longer duration of disease (31 vs. 10 or 14 months) and a lower mean BMI (13.5 vs. 14.7 and 15.4 kg/m^2^). These three studies found the same risk factors as ours, with a positive correlation between admission BMI z-scores, and admission clearance values. However, some studies included other risk factors such as lowered heart rate or low potassium levels at admission.

The other three studies used other equations to calculate eGFR and found mean eGFR values of 83, 99.8 and 86 ml/min/1.73 m^2^ [10, 18, 25]. The authors found no association between baseline eGFR and baseline BMI, but the equations used are not reliable for assessing renal function in this population [24].

In adult populations (age > 18 years), the glomerular filtration rate evaluation is also very heterogeneous across different studies, with a mean measured GFR (24 h urinary creatinine excretion) for Boag [26] and Takakura [27] at respectively 50 ml/min and 86.3 ml/min, a 51Cr-EDTA measure for Delanaye [28] at 67.5 ml/min and a mean eGFR calculated with Cockroft and Gault equation for Fabbian [29] at 80 ml/min.

Despite this problem of homogeneity in the evaluation of the glomerular filtration rate, all studies reported impairment of renal function. Our study shows a very large proportion of patients with an eGFR below 90 ml/min, reaching over 80%, which is much higher than in other published studies. In adolescent studies, proportions were 33, 35, 37 and 45% respectively for Downey, Trahan, Gurevich and Stheneur [10, 12, 14, 24]. In adult populations, only one study explicitly reports the proportion, finding that 100% (out of 10 participants) had an eGFR below 90 ml/min [26]. The proportion of low eGFR found in our study is therefore probably related to the scale of malnutrition in our population, as the level of malnutrition is lower than in previous published studies (whether among adolescents or adults).

The factors that influence the extent of renal damage are debated in the literature [30]. In bivariate analysis, we found 6 factors related to renal damage: BMI at admission, duration of disease, number of hospitalizations, and treatment with psychotropic drugs (neuroleptics or hypnotics or benzodiazepines); all these factors are linked to the severity of AN in terms of nutrition (state (BMI), duration of illness, treatment trajectory (indication of inpatient treatment), and comorbidity with psychiatric disorders (reflected by psychotropic medication) [31]. However only the admission BMI explained renal damage in the multivariate analysis. We therefore believe that undernutrition in itself could play a role in kidney function: the lower the BMI, the more likely is damage to the kidney. Undernutrition is the predominant factor in the severity of renal impairment.

Similarly, previous studies have shown the same link among both adolescents [11, 12], and adults [26, 32]. This result is important because it suggests that the kidney is sensitive to undernutrition, regardless of the pathophysiological mechanism that underlies this damage. The extent of bradycardia, often found in this population, correlated with the extent of malnutrition, is a possible explanation for the impact of malnutrition on the kidney. With reduced perfusion, the kidney suffers and its function deteriorates. For some patients, there is also fluid restriction, particularly among younger patients, increasing kidney damage.

One of the other two hypotheses concerning the factors possibly involved is chronic dehydration, frequently found among patients with AN, leading to intravascular volume depletion [27]. The second hypothesis concerns chronic hypokalemia resulting from self-induced vomiting or diuretics/ laxative abuse [27, 33]. While these two factors seem important, not all studies found these factors to be determining for the extent of renal damage. Downey et al. [14] and Rosen et al. [25], who studied fluid movement during refeeding in restrictive eating disorder, showed that the improvement in renal function could not be attributed to increased renal perfusion from rehydration since a similar change in serum creatinine was observed irrespective of whether participants had severe or minor early renal fluid losses. Also some studies, in line with our results, did not find any association between vomiting, laxative use, and renal impairment [11, 12, 24]. It is likely that prolonged hypokalaemia leads to nephrocalcinosis and kidney damage after a significant period of time below normal levels. In our study, patients with the AN-BP subtype had a lower BMI than those with AN-R subtype, so the importance of undernutrition could have taken precedence over the influence of kalaemia.

Finally, according to Fohlin [34], calorie deficiency leads to decreased renal function through an adaptive mechanism, whereby the fall in GFR could enable a reduction in energy expenditure. But this would not explain the maintenance of low glomerular filtration despite nutritional rehabilitation.

Even though all studies highlight an improvement in renal function with refeeding, some participants still presented varying degrees of impairment, which seemed to worsen with the severity and recurrences of undernutrition, leading to long-term kidney dysfunction with a risk of terminal renal failure in adulthood [13, 16, 35–37].

### Strengths and limitations

The strengths of this study are, first, the size of the study sample and the severity of AN. With nearly 200 participants included, hospitalized for AN with a mean z-score BMI of − 3.6, this study clearly shows the prominence of renal impairment in severe AN. Second, the fact that the study was multicentre also makes it possible to consider that the results obtained are generalizable to all units managing these patients across age groups in France.

The reason for hospitalisation for clinicians is AN, even if strict criteria can classify a third of the patients as sAN. We chose to include all patients because the risk of kidney damage does not appear to be any lower for patients with sAN.

On the other hand, one of the limitations is the possibility of under-reporting of medication, in particular laxatives or diuretics. Another could be that the biological assays were all conducted at one time and thus do not reflect the chronicity of the disturbances. In particular, we did not investigate the possibility of chronic hypokalaemia, often suspected to be a factor in renal damage. With so many subjects studied, and so many centres involved, the GFR was calculated and not evaluated, but the correlation between the Cockroft and Gault formula and GFR has already been shown in this type of population [24].

## Conclusion

In periods of acute undernutrition, it is essential to assess renal function, especially given that the dosage of certain medications needs to be adapted, and fluid intake should be adjusted, as should the daily amounts of protein and salt.

These elements are rarely taken into account in the management of hospitalized AN patients, although the risk of permanent renal damage is high. Therefore, clinicians should thoroughly assess renal function in cases of undernutrition in the implementation of their short and longer-term care plans.

## Data Availability

The data that supports the findings of this study is available from the corresponding author upon reasonable request.

## Abbreviations

AN: Anorexia nervosa
CKD: Chronic kidney disease
eGFR: Estimated Glomerular filtration rate
SD: Standard deviation
